# New species of *Xiphoscelis* Burmeister, 1842 (Coleoptera, Scarabaeidae, Cetoniinae) from arid regions of South Africa and Namibia

**DOI:** 10.3897/zookeys.879.37721

**Published:** 2019-10-09

**Authors:** Renzo Perissinotto, Petr Šípek

**Affiliations:** 1 School of Environmental Sciences, Nelson Mandela University, P.O. Box 77000, Port Elizabeth 6031, South Africa Nelson Mandela University Port Elizabeth South Africa; 2 Department of Zoology, Charles University, CZ- 128 44 Vinicna 7, Praha 2, Czech Republic Charles University Prague Czech Republic

**Keywords:** Afrotropical region, fruit chafers, identification key, immature stages, life cycle, Succulent Karoo, taxonomy, Xiphoscelidini

## Abstract

Two new species of the southern African genus *Xiphoscelis* Burmeister, 1842 are recognised and described, *X. braunsi***sp. nov.** from the Eastern and Western Cape Karoo (South Africa) and *X. namibica***sp. nov.** from the Huns Mountains of southern Namibia and adjacent ranges in South Africa. These were previously overlooked and grouped together with *X. schuckardi* Burmeister, 1842, but further material and more in-depth analyses have now revealed their clear separation on the basis of key diagnostic features, including clypeal structure, metatibial spur development and aedeagal shape. The densely and coarsely costate elytral structure and the black to brown colour of these species are symplesiomorphies shared with a number of the most primitive genera among the African Cetoniinae. However, these characters also reflect the convergent adaptation to hot and arid conditions they share with several other species occurring in this region. Phylogenetic relationships of the genus with other Cetoniinae are explored using the larval characters highlighted in the description of the 3^rd^ instar larva of *X. braunsi***sp. nov.** The extraordinary hypertrophy observed in the male metatibial spur of species in this genus, and particularly in *X. schuckardi*, appears to represent a defence mechanism against potential predators on the ground, apart from playing a role during mating.

## Introduction

The genus *Xiphoscelis* has been recognised since its first description as characterised by unique features, such as a narrow mesometasternal process, enlarged metafemur, low subhumeral elytral arch, round pronotum and regularly costate elytra. To accommodate these, apparently plesiomorphic features, a supra-generic grouping was proposed with the aim of clustering together a variety of genera sharing the key characters. Burmeister (1842) suggested the name Xiphoscelideae, which was later converted to Xiphoscelidini by Schenkling (1921), in order to delineate a new tribal subdivision.

Krikken (1984) made substantial amendments to this tribe, by transferring several genera into or outside it, relying heavily on the lack/presence of mesometasternal protrusion and the degree of approximation of the mesocoxae. Apart from the proper Afrotropical genera, Krikken (1984) also included a few genera from the Madagascan subregion (i.e., *Scheinia* Ruter, 1957 and *Plochiliana* Ruter, 1978) and Australia (i.e., “Genus 1 (= *Pseudoclithria* auctorum” and other yet unnamed genera at that stage, Krikken 1984: 46–47). This was still largely reflected in the iconographic monograph of Sakai and Nagai (1998).

However, later Holm and Marais (1992) proposed the removal of most of the Afrotropical genera included by Krikken (1984), leaving only the genus *Xiphoscelis* and its immediate “precursors” (*Protoclita* and *Ischnostomiella*) in it, and the downgrade of the tribe to a subtribe. More recently, Beinhundner (2017) has included the following among the Afrotropical (excluding Madagascar) genera within this tribe: *Aporecolpa* Lansberge, 1886; *Ischnostomiella* Krikken, 1978; *Myodermidius* Bourgoin, 1920; *Protoclita* Krikken, 1978; and *Xiphoscelis*.

As already pointed out by Krikken (1984), it is obvious that this supra-generic cluster is among the most uncertain of all the subdivisions currently recognised in the taxonomy of Cetoniinae. In his insightful account, this author articulated the following possibility: “A synapomorphy for the entire group is not available, and it may well be that most of the included groups (or even all of them) stand phylogenetically at the base of other tribes, which could ultimately lead to a complete dissolution of the tribe Xiphoscelidini” (Krikken 1984).

To complicate matters further, recent molecular analyses indicate that the phylogeny of Cetoniinae in general needs a substantial revision, as large incongruences with the traditional concepts are emerging (Šípek et al. 2016). This applies to all the major classification systems proposed to date, namely those of Schenkling (1921), Krikken (1984) and Holm and Marais (1992).

There are currently three species described within the genus *Xiphoscelis*, two of which (*X. lenxuba* Perissinotto, Villet & Stobbia, 2003 and *X. sneeubergensis* Perissinotto, Villet & Stobbia, 2003) were only recently separated from the type and only species previously recognised, *X. schuckardi* (Perissinotto et al. 2003, Beinhundner 2017). Further analyses of type specimens and availability of new material and data have now revealed that another two species need to be erected in order to account for the differences observed in the populations that were collectively grouped under *X. schuckardi* by Perissinotto et al. (2003). These are described here, along with an update of the biology of the genus and the first detailed description of the 3^rd^ instar larval stage of a species (*X. braunsi* sp. nov.) within the genus.

## Materials and methods

Holotype specimens of both *Xiphoscelis schuckardi* Burmeister, 1842 (♂, 17 mm total length, “Pr. b. sp. Sch.”) and *Xiphoscelis gariepena* Schaum, 1849 (nec Gory & Percheron) (♀, 15.8 mm total length, “Afr. Austr”) were studied in detail through high-resolution photographs kindly provided by Karla Schneider (MLUH) and Giulio Cuccodoro (MHNG), respectively.

Other specimens were obtained through field collections during the period 1995–2018 (R Perissinotto & L Clennell legit), or from museum and private collections (as per list provided below). Fresh specimens were either caught in flight using standard nets after rainfall events, excavated from underground or obtained after rearing third instar larvae collected in the wild under laboratory controlled-conditions. In the laboratory, larvae were kept in plastic containers of 1–5 L capacity, containing the natural soil and detrital material found in situ. Water was sprayed at the soil surface at regular intervals of about 1–2 weeks until pupation.

Data on distribution, period of adult activity and other biological information for all the species of the genus *Xiphoscelis* were also obtained from Péringuey (1907), Holm and Marais (1992), Sakai and Nagai (1998), Perissinotto et al. 2003 and Beinhundner (2017). The key geographic abbreviations used within the text are as follows: NAM = Namibia; WC = Western Cape Province (South Africa); NC = Northern Cape Province (South Africa); EC = Eastern Cape Province (South Africa).

As in previous works, the description of adult morphological characters follows the terminology of Krikken (1984) and Holm and Marais (1992). Specimen total length and maximum width were measured using a Vernier calliper, from the anterior margin of the clypeus to the apex of the pygidium and at the widest point of the elytra, respectively. Photos of specimen dorsal, ventral and lateral views were taken with a Nikon CoolPix S9700 digital camera with macro setting, while higher-resolution photos of specimen’s clypeus, pygidium and male genitalia were obtained using a Nikon DigitalSight DS–Fi2 camera attached to a Nikon SMZ25 dissecting microscope. The background was removed from the photos using Microsoft Word 2010 (Picture Tools), in order to increase clarity of resolution. The Combine ZP Image Stacking Software by Alan Hadley (alan@micropics.org.uk) was used to obtain z-stacked composite images.

The identity of the larvae was confirmed by both rearing specimens to adulthood and by molecular match (COI) with adult specimens. Specimens for DNA extraction were stored in 96% ethanol immediately after capture. Genomic DNA from thoracic leg muscle tissue (both larvae and adults) was extracted non-destructively using a Qiagen Blood and Tissue Kit, following standard protocols. Voucher specimens are deposited at the NMCR. Partial sequences of the mitochondrial protein coding gene cytochrome oxidase subunit 1 (Cox1) were used in the study. For a detailed description of the laboratory protocol see Vondráček et al. (2018). The larval sequences proved to be identical to those of the adults (100% match). The respective sequences will be submitted to the GenBank (NCBI) together with those of other Cetoniinae once the ongoing investigation of the phylogenetic relationships of South African Cetoniinae has been finalized.

Larval material was examined with an Olympus SZ9 and a Nikon SMZ 745 stereomicroscope, under which measurements were taken with an ocular grid. Habitus photographs were taken using a Canon EOS 70D camera fitted with Canon EF–S 60 mm f/2.8 Macro USM lens or Canon MP–E 65 mm f/2.8 1–5× macro lens. Microscopic slides were photographed using a Canon EOS 70D camera mounted on an Olympus SZ9 stereomicroscope. Partially-focused images of specimens were combined using Zerene Stacker (Zerene Systems LLC, Richland, USA). Structures examined using scanning electron microscopy (JEOL, Model 6380, Tokyo) were cleaned in 10% lactic acid for 24 h and submerged into a Sonorex ultrasonic bath (Bandelin electronics, Berlin) for 30 s, dried in a heating chamber, or using critical point drying, and mounted on aluminium plates. All pictures were digitally enhanced using Adobe Photoshop CC.

The terminology for larval description follows Böving (1936), Ritcher (1966) and Sawada (1991). Antennomeres I–IV were labelled with the respective abbreviations (an I–an IV). In order to give the most accurate information on chaetotaxy, hair-like setae of the cranium and other structures were classified by their relative size into two groups: medium to long (80–300 μm) and minute to short (5–40 μm or less).

To compare the observed larval morphological characters of *X. braunsi* sp. nov. with those of other cetoniines from various clades, a basic phylogenetic analysis based on a matrix of 77 larval morphological characters of 13 taxa was performed, as modified from Kouklík (2017) (Appendix 1). A heuristic parsimony analysis was carried out with PAUP version 4.0b10 (Swofford 2002), using 1000 random taxon additions and tree bisection-reconnection branch swapping. Missing data were coded with a question mark (?) and inapplicable characters as ‘en’ dash (–). The data matrix was prepared using Nexus data editor software and branch support was assessed by bootstrapping 1000 randomly selected trees (Felsenstein 1985). The TreeView and Winclada programs (Nixon 2002) were used to visualise the trees and character state optimisation.

Specimen repositories are abbreviated as follows:

**BMPC** Jonathan Ball and Andre Marais Private Collection, Cape Town, South Africa

**ISAM** Iziko South African Museum, Cape Town, South Africa

**MLUH** Martin Luther Universität Zoologische Sammlung, Halle, Germany

**MHNG** Muséum d‘Histoire Naturelle, Genève, Switzerland

**NMCR** Národní Museum, Prague. Czech Republic

**SANC** South African National Collection of Insects, Pretoria, South Africa

**SRPC** Sébastien Rojkoff Private Collection, Sourcieux les Mines, France

**TGPC** Thierry Garnier Private Collection, Montpellier, France

**TMSA** Ditsong National Museum of Natural History (formerly Transvaal Museum), Pretoria, South Africa

**UCTC** University of Cape Town Entomological Collection, Cape Town, South Africa

**UKCR** Univerzita Karlova, Katedra Zoologie, Prague, Czech Republic

**ZMHB** Museum für Naturkunde der Humboldt Universität, Berlin, Germany

## Taxonomy

### Xiphoscelis braunsi

Taxon classificationAnimaliaColeopteraScarabaeidae

Perissinotto & Šípek sp. nov.

1D912D81-4297-579D-BC90-04587D162CCA

http://zoobank.org/C4DB98B6-5EC0-4E16-8E00-87232F59E100

Figs 1 , 4 , 8 , 9

#### Diagnosis.

**Figure 1.**
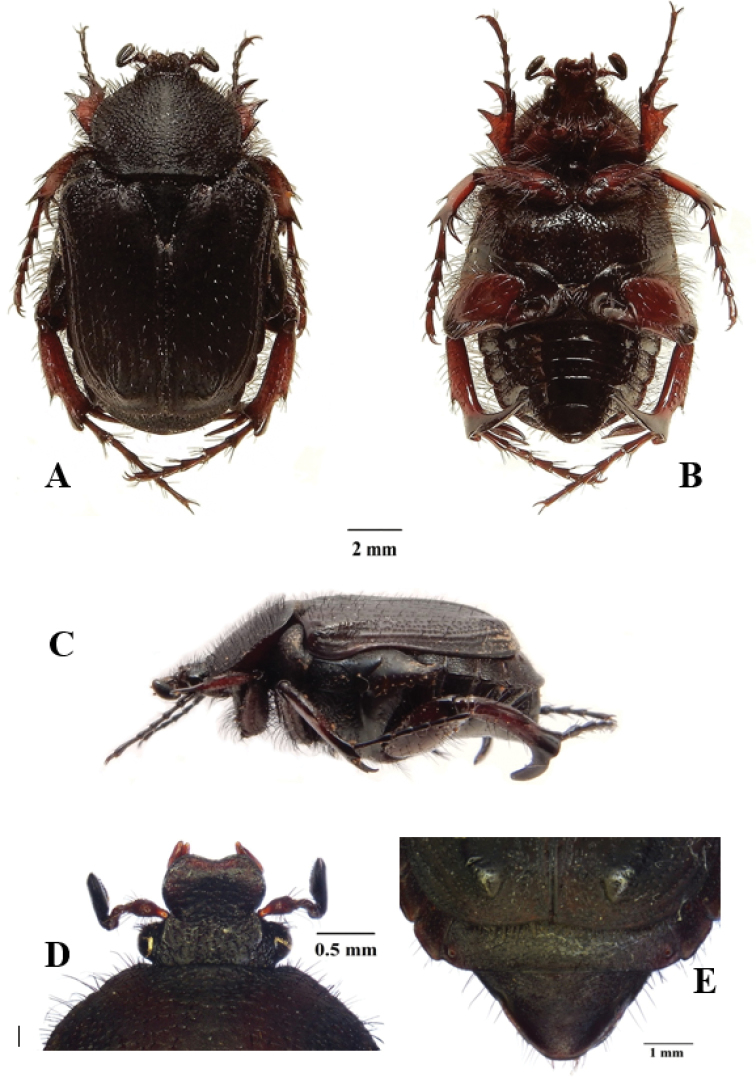
*Xiphoscelis braunsi* sp. nov., male: dorsal (**A**), ventral (**B**) and lateral (**C**) habitus; clypeus (**D**) and pygidium (**E**). Photographs by Lynette Clennell.

This species differs from *X. schuckardi* by its matte, black to brown dorsal colouration (black and shiny in *X. schuckardi*) and the elytral costae which are weakly elevated and poorly visible, rather than prominent as in *X. schuckardi* (Figs 1, 3). The dorsal sculpture of *X. braunsi* is also scattered and shallow, in contrast to that of *X. schuckardi*, which is generally substantially denser and deeper. In *X. braunsi*, the anterior clypeal margin is deeply sinuate and its lateral margins smoothly rounded (Fig. 1D). In *X. schuckardi*, the anterior margin is moderately sinuate while the lateral margins are rather straight to arcuate (Fig. 3D). The total body length of *X. braunsi* falls within the range of 11–16 mm (*N* = 21), while *X. schukardi* normally exhibits a larger size of 14–22 mm (*N* = 26).

Finally, the parameres of the two species are also different at the level of the inner apical end of the dorsal lobes, which is finely pointed in *X. braunsi*, but rather blunt in *X. schuckardi* (Figs 4, 6). The entire apical surface of the dorsal lobes is covered in relatively long setae in *X. braunsi*, while these are extremely short and barely visible in *X. schuckardi*. The diagnostic differences between *X. braunsi* and *X. namibica* are highlighted in the section below, under the description of the latter species.

#### Description of holotype male.

(Figs 1A–E, 4A–C) ***Size.*** Length 14.8; width 7.7 mm.

***Body.*** Black to dark brown, completely matte except for small worn ridges on elytral umbones (Fig. 1A); head and pronotum disproportionally small relative to abdomen size; metacoxa, abdominal sternites and pygidium protruding remarkably outside elytral margins; exhibiting scattered and shallow sculpture throughout dorsal surface associated with short to medium dark setae, becoming longer and denser on antero-lateral margins (Fig. 1A, C, D).

***Head.*** Black to dark brown, with round sculpture on frons, becoming irregularly shaped on vertex; ultrafine rugosity across entire surface; long, erect black setae restricted to eye canthus and antennal pedicel; clypeus markedly bilobate and deeply concave, with anterior margin sharply elevated and lateral margins perfectly rounded both posteriorly and anteriorly; antennal clubs and flagellum black to dark brown, of normal cetoniine length; pedicel dark brown, becoming lighter towards base.

***Pronotum.*** Dark brown and matte, becoming blackish at margins; smoothly rounded at all margins except antero-lateral, which exhibit sharp angles leading to medio-apical, weakly elevated transversal protuberance; posterior margin forming straight line in front of scutellum; small, scattered round to horse-shoe punctures throughout surface, becoming denser towards lateral and anterior margins; thick, black, medium to long setae regularly distributed across entire surface and emerging at centre of punctures, becoming denser and longer at lateral margins (Fig. 1A, C).

***Scutellum.*** Black on sides to dark brown on disc; isoscelic triangular with sharply pointed apex and deep but narrow lateral grooves; with regularly spaced horse-shoe punctures restricted to basal and baso-lateral margins; dense line of short, light setae along basal margin, just below posterior pronotal margin (Fig. 1A).

***Elytron.*** Black to dark brown, not covering entire abdomen and leaving external projection of sternites and pygidium partly exposed; subhumeral arch very low and postero-apical declivity extremely steep and abrupt; with costae 1–4 poorly elevated in basal two-thirds, becoming virtually flat in apical third; 5^th^ costa and umbones raised, with latter showing shiny area on top; rest of surface matte and regularly sculptured with geminate striae along intercostal spaces 1–4, becoming round to horse-shoe beyond 5^th^ costa; with sparse short, but thick and erect black setae across whole surface, except umbones; apex without spinal projection and weakly rounded (Fig. 1A, B).

***Pygidium.*** Black at base gradually becoming dark brown towards apex; remarkably narrow and triangular in shape, with basal and baso-lateral margins sharply upturned; with dense, rugose sculpture on basal third, becoming scattered and round towards apex; with moderate central bulge and shallow, symmetric baso-lateral depressions; without pubescence on general surface but with lining of black, long setae along entire apical margin (Fig. 1E).

***Legs.*** Tarsal segments consistently black and elongate, but tibiae reddish-brown with black tips and of normal cetoniine thickness and length; protibia tridentate, with proximal tooth drastically reduced; mesotibia bearing mid outer spine with four thick setae on its surface, two apical spines and two spurs of small to medium size; metatibia with extreme hypertrophic inner spine and spurs, inner spine 2–3 times as thick and 1.5 times as long as spurs; (Fig. 1A, B, C); femora reddish-brown with black edges and bearing long, thin setae; pro- and meso-femora of normal size, but metafemora hypertrophic.

***Ventral surface.*** Shiny, reddish-brown to black; with thin and long dark setae on prosternum, coxae and all femoral margins, becoming shorter and more sparse on other surfaces, particularly abdominal sternites; all setae emerging at centre of small and round sculptures; mesometasternal process extremely small, not protruding forward or upwards and partly covered by coxal bases, reddish-brown to black, with scattered round punctures and thin setae on surface; abdominal sternites initially flat, but forming concavity at middle particularly in area of sternites 5–7.

***Aedeagus.*** Parameres with dorsal lobes tapering gradually and smoothly towards apex, forming short spinal apical inner end, covering completely ventral lobes in dorsal view (Fig. 4A); exhibiting long, thin setae along entire apical margin (Fig. 4 A–C); apical surface rounded-triangular in frontal view (Fig. 4C).

***Derivatio nominis.*** This species is dedicated to the memory of Hans Brauns (1857–1929), renowned physician and entomologist, who during the early 20^th^ century lived and collected extensively in the Willowmore District of the Eastern Cape Province. Most of the early specimens of the new species described here formed part of his collection.

#### Description of female.

The only reliable and consistent external feature of sexual dimorphism in this species lies in the development of the metatibial internal apical spine and spurs, which are far less hypertrophic in the female, compared to the male. Also, like in most cetoniines, the protibiae and protarsi are appreciably shorter in the female than in the male, and the abdominal sternites of the male are usually concave in the central area while those of the female tend to be flat or slightly convex.

#### Distribution.

This species appears to be restricted to the Eastern and Western Cape provinces of South Africa. Apart from the long series collected at Willowmore by Brauns in the early part of the 20^th^ century, specimens have recently been found in mountainous areas of the western part of the Eastern Cape and in the interior regions of the Western Cape, at altitudes > 500 m but not exceeding 1000 m asl. Thus, the species appears to follow the geographic range of the Cape Fold Belt, where it inhabits the lower slopes of its mountain ranges (Fig. 7).

#### Biology.

Both literature and specimen data records report regular occurrences of association of this species with the southern harvester termite, *Microhodothermes viator* (e.g., Holm and Marais 1992, Perissinotto et al. 2003, Hans Brauns data labels). In particular, numerous larvae were collected recently at Worcester (Western Cape), from frass accumulations of *M. viator* that constructs heuweltjies, and reared successfully in the laboratory (Mike Picker, pers. comm.). However, this does not appear to be an obligatory or even predominant association, as most available records and observations are actually of a different nature. Both adults and larvae have most often been found in or around shrubs of a variety of karooid plants, like renosterbos, *Dicerothamnus rhinocerotis*, or *Psilocaulon* sp. (RP pers. obs., Petr Malec pers. comm.).

#### Remarks.

While the ventral habitus of this species is remarkably stable in colour, being predominantly reddish-brown, the dorsal surface ranges across two extreme varieties, one completely black (Fig. 9) and the other reddish-brown (Fig. 8). All the variations between these two extremes have been observed, with the populations at the eastern end of the distribution range normally showing a dominance of light forms and the westernmost populations exhibiting predominantly black habitus. Within the type series analysed in this study, the size ranges as follows: ♂ length 11.1–16.0 mm, width 5.3–8.5 mm (*N* = 13); ♀ length 12.3–15.6 mm, width 5.9–8.3 mm (*N* = 8).

#### Type material.

**Holotype** (♂): South Africa, EC, Fullerton, 5 Jan 2017, R. Perissinotto & L. Clennell (ISAM). Paratypes: 1 ♂, Beauf. W., 1883, (ISAM: COL–A026571); 1 ♂, Algoa Bay, Capland, Dr Brauns (TMSA: CPH7888); 1 ♂, Capland, Willowmore, 1 Nov 1919, Dr Brauns (TMSA: CPH7883); 2 ♀, Capland, Willowmore, Jan 1916, Dr Brauns, *Hadotermes viator* Latr. (TMSA: CPH7882); 4 ♂, 1 ♀, Kliplaat, Capland, Dr Brauns, Jan 1911 (TMSA: CPH7889); 2 ♂, Willowmore, Capland, Dr Brauns, Jan 1909 (TMSA: CPH7881); 1 ♂, Willowmore, Capland, Dr Brauns, Jan 1909, *Xiphoscelis rufa*, J. Krikken ms 1985 - Paratype, *Xiphoscelis gariepina* G & P. (TMSA); 1 ♂, Willowmore, Capland, Dr Brauns, Jan 1909, *X. Gariepena* G. & P., Cum typo comp. (TMSA); 1 ♀, Willowmore, Capland, Dr Brauns, 20 Nov 1902 (TMSA: CPH7880); 1 ♂, 2♀, No data (TMSA: CPH7885); 1 ♂, Willowmore, Capland, Dr Brauns, Jan 1909, *Xiphoscelis gariepena*, C. *Hadotermes viator* Haq., coll. Jul. Moser (ZMHB); 1♂, Willowmore, Capland, Dr Brauns, 20 Nov 1902 (ZMHB); 1♀, Pr. b. sp. Meyer, coll. Nonfried Africa orient., coll. Jul. Moser (ZMHB); 1 ♂, 1 ♀, Willowmore District, Dec 1960, Dr Brown (BMPC); 1 ♂, Matjesfontein (C. C.), E. Simon 1893 (TMSA: CPH7886); 1 ♀, 5 May 1965 (TMSA: CPH7884); 1 ♂, Three Sisters, CP. RSA, 26 Oct 1973, N. J. Duke (TMSA: CPH7887); 1 ♂, South Africa, Western Cape, Matjiesfontein, 33°13'S, 20°35'E, Purcell leg. (SANC: COLS–12182); 1 ♂, South Africa W. Cape, Anysberg N. Res. 29 Oct 96, C. Price (BMPC); 1 ♀, South Africa E. Cape, Nr. Willowmore, 24 Dec 99, R. Perissinotto & L. Clennell (BMPC); 1 ♀, South Africa, EC, Antoniesberg, 23 Dec 2002, R. Perissinotto & L. Clennell (BMPC); 3♂, 1♀, South Africa, WC, Garcia Pass, 20 Jan 2016; 6♂, 2♀, South Africa, EC, Fullerton, 5 Jan 2017, R. Perissinotto & L. Clennell; 2♂, 2 ♀, South Africa, EC, Nr. Streytlerville, 18 Jun 2016, R. Perissinotto & L. Clennell; 5♂, 4♀, South Africa, EC, Sarah Baartman District (Dr Beyers Naudé Municipality), 15 km NW of Willowmore, 870 m, 5.I.2017, Malec & Šípek leg. (PMPC, PSPC); 1♂, 1♀, South Africa, Eastern Cape, Willowmore env., 3–4.I.2017, P. Malec & P. Šípek leg. (PSPC, NMCR: XS061RSA_1); 1♀, South Africa, Eastern Cape, Willowmore env., 3-4.I. 2017, P. Malec & P. Šípek leg., Ex larva bred from wild larvae, P. Malec breeding; unspecified no. of specimens, South Africa (WC), Worcester, Sep 2017 [common in frass accumulations of *Microhodotermes viator* termites, heuweltjies] (Mike Picker, pers. comm.).

### Xiphoscelis namibica

Taxon classificationAnimaliaColeopteraScarabaeidae

Perissinotto sp. nov.

AB3593B9-3FD6-5B54-B14E-C6912663BE05

http://zoobank.org/FA69288D-39FB-44C3-B900-E62AC514097F

Figs 2 , 5

#### Diagnosis.

**Figure 2.**
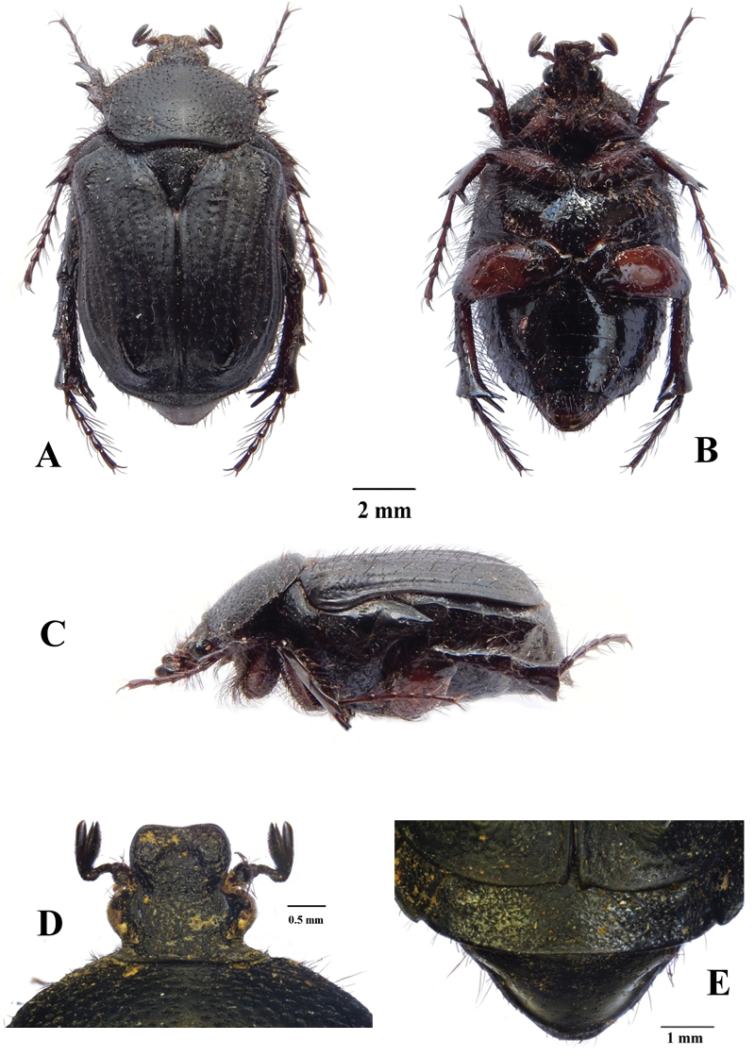
*Xiphoscelis namibica* sp. nov., male: dorsal (**A**), ventral (**B**) and lateral (**C**) habitus; clypeus (**D**) and pygidium (**E**). Photographs by Lynette Clennell.

**Figure 3.**
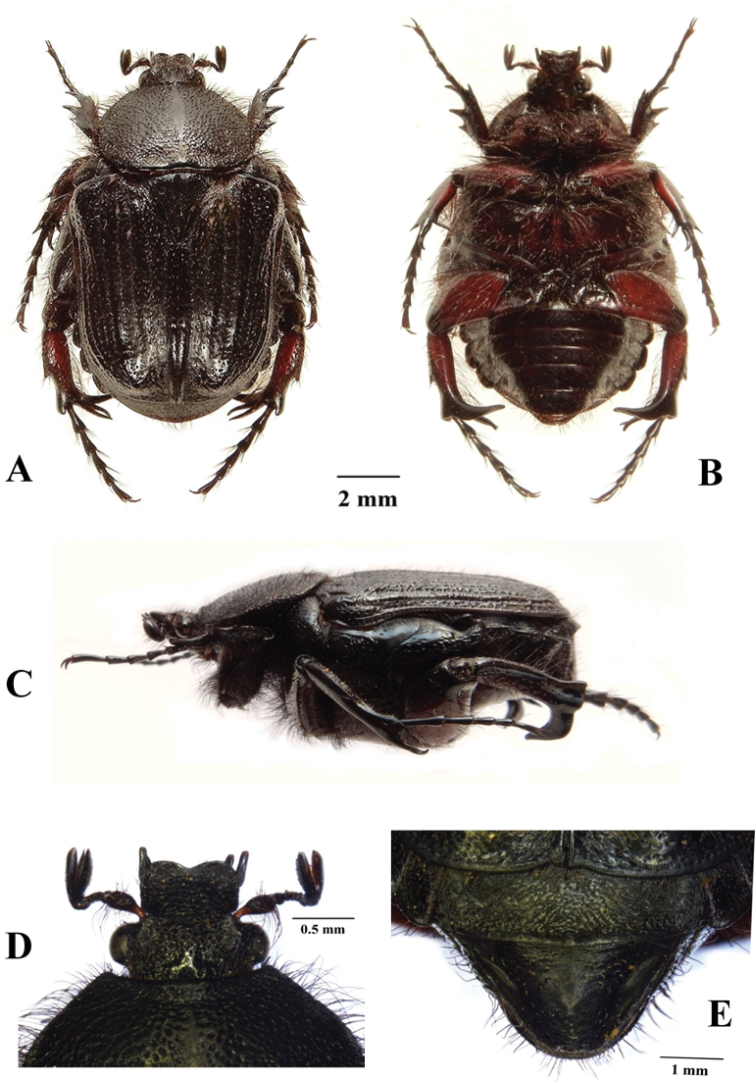
*Xiphoscelis schuckardi* Burmeister, 1842, male: dorsal (**A**), ventral (**B**) and lateral (**C**) habitus; clypeus (**D**) and pygidium (**E**). Photographs by Lynette Clennell.

*Xiphoscelis namibica* can best be separated from both *X. schuckardi* and *X. braunsi* by the characteristics of its parameres, as it is the only species among the three to exhibit an apical protuberance on the inner margin of each dorsal lobe (Fig. 5A–C). There are also external morphological characters that can be used in the diagnosis of this species, the most prominent of which are clypeal shape and the size of the metatibial spurs. Unlike in the other two species, in *X. namibica* the anterior margin of the clypeus ranges from being weakly sinuate to straight, while the lateral margins are arcuate like those observed in *X. schuckardi* (Fig. 2D). The metatibial spines and spurs are generally hypertrophic in the genus *Xiphoscelis*, with the internal spine normally exceeding the thickness and length of the spurs by several fold and attaining extreme disproportions in males (Perissinotto et al. 2003). In *X. namibica*, however, the metatibial spurs are as long as the inner spine in both sexes and neither of them reaches lengths comparable to those observed in the other two species under comparison (Figs 1–3).

#### Description of holotype male.

(Figs 2A–E, 5A–C) ***Size.*** Length 14.3; width 8.0 mm.

***Body.*** Completely black and matte, except for small worn ridges on elytral umbones (Fig. 2A); head and pronotum of normal proportions with respect to abdomen size; metacoxa, abdominal sternites and pygidium moderately protruding outside elytral margins; with scattered and shallow sculpture throughout dorsal surface associated with short to medium dark setae, becoming longer and denser on antero-lateral margins (mostly fallen or broken due to specimen being retrieved from the field sometime after death, Fig. 2A, C, D).

***Head.*** Entirely black, with coarsely round sculpture throughout surface; ultrafine rugosity across entire surface; long, erect black setae on eye canthus and antennal pedicel; clypeus weakly bilobate but deeply concave, with both anterior and lateral margins equally elevated, lateral margins smoothly rounded all around; antennal clubs, flagellum and pedicel black and of normal cetoniine length; pedicel becoming lighter and brown towards base.

***Pronotum.*** Black and matte; regularly round in shape, except at antero-lateral margins, where sharp angles lead to medio-apical, moderately elevated transversal protuberance; posterior margin forming perfectly straight line in front of scutellum; round punctures regularly spaced across surface, but becoming more scattered on disc and denser at margins and on lateral declivities; thick, black, setae of medium length visible only on lateral margins (Fig. 2 A, C).

***Scutellum.*** Completely black; isoscelic triangular with weakly rounded apex and lateral grooves absent on basal third but well developed along other two-thirds towards apex; with dense but irregularly shaped punctures on basal and baso-lateral margins, but absent on central part of disc and on apical third; few, thick and black erect setae scattered across basal third of surface (Fig. 2A).

***Elytron.*** Completely black and matte, narrower than abdomen leaving apical projection of sternites and pygidium partly exposed; subhumeral arch very low and postero-apical declivity extremely steep and abrupt; all costae subequally and weakly elevated, with 5^th^ and 6^th^ costae and umbones raised; surface densely sculptured with geminate striae or round to irregular punctures along intercostal spaces, becoming very sparse and occasional on surface of costae; sparse short, but thick and erect black setae across whole surface, except umbones; apex with short but distinct spinal projection (Fig. 2A, B).

***Pygidium.*** Completely black, narrow and broadly triangular in shape, with basal and lateral margins sharply upturned; with uniformly sparse horse-shoe sculpture across surface; convex with small baso-lateral depressions; without pubescence on general surface but with lining of black, long setae along lateral and apical margins (Fig. 2E).

**Figure 4.**
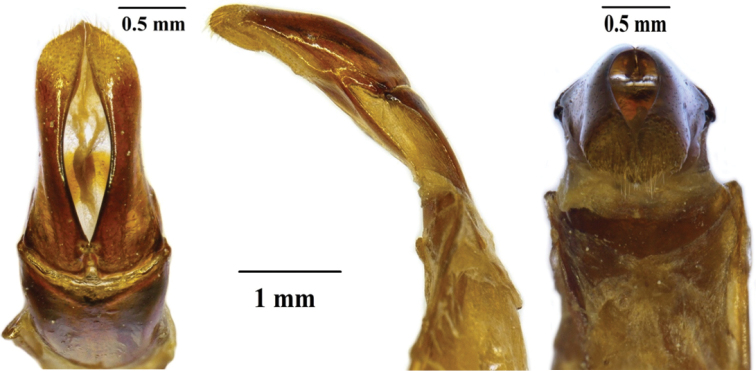
*Xiphoscelis braunsi* sp. nov. (WC, Garcia Pass): dorsal (**A**), lateral (**B**) and frontal (**C**) views of aedeagus. Photographs by Lynette Clennell.

***Legs.*** All legs black in dorsal view, with tarsal segments elongate, but tibiae of normal cetoniine thickness and length; protibia tridentate, with proximal tooth reduced and other two teeth severely worn; mesotibia exhibiting mid outer spine, two apical spines and two spurs of small to medium size; metatibia with slightly hypertrophic inner spine and spurs, inner spine much thicker but shorter than spurs (Fig. 2A–C); femora reddish-brown at base, becoming black distally and at joints, bearing long, thin dark setae; pro- and meso-femora of normal size, but metafemora hypertrophic.

***Ventral surface.*** Shiny and black, with reddish-brown areas restricted to part of coxae and basal portion of femora; with thin and long dark setae on prosternum, coxae and all femoral margins, becoming shorter and more sparse on other surfaces, particularly abdominal sternites; all setae emerging at centre of small and round sculptures; mesometasternal process extremely small, not protruding forward or upwards and partly covered by coxal bases, black and with scattered round punctures and thin setae on surface; abdominal sternites slightly convex, becoming flat at middle particularly in area of sternites 5–7.

**Figure 5.**
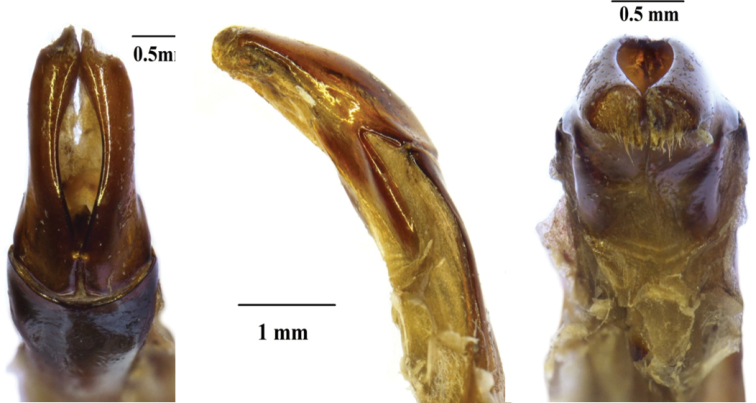
*Xiphoscelis namibica* sp. nov. (NAM, Namuskluft): dorsal (**A**), lateral (**B**) and frontal (**C**) views of aedeagus. Photographs by Lynette Clennell.

***Aedeagus.*** Parameres with dorsal lobes tapering abruptly towards apex, forming steeply elevated apical spine at inner end, covering completely ventral lobes in dorsal view (Fig. 5A); exhibiting long, thin setae along entire apical margin (Fig. 5A–C); apical surface approximately circular in frontal view (Fig. 5C).

**Figure 6.**
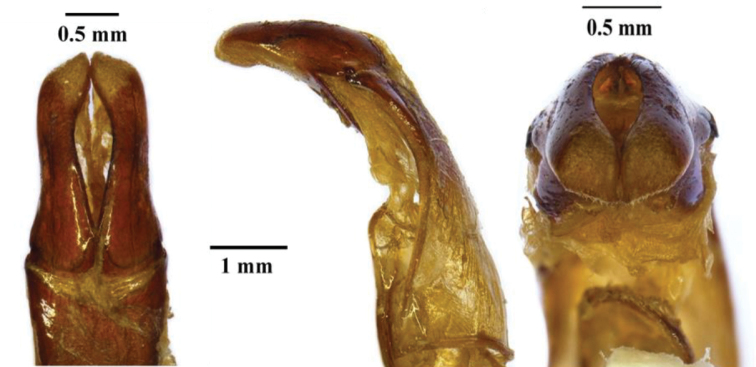
*Xiphoscelis schuckardi* Burmeister, 1842 (NC, Wallekraal): dorsal (**A**), lateral (**B**) and frontal (**C**) views of aedeagus. Photographs by Lynette Clennell.

***Derivatio nominis.*** With the exception of one female from O’Kiep (South Africa), so far, all the specimens known for this species and representing the type series originate from the same locality, in south-western Namibia. Hence the obvious geographic link to its name.

#### Description of female.

Unlike with all the other species of the genus, it is virtually impossible to separate the two sexes of *X. namibica* on the basis of external morphology alone. This is because the metatibial internal apical spine and spurs of its male (Fig. 2A–C) are just as poorly developed as those of the female: a truly unique situation within this genus, with essentially no detectable sexual dimorphism. The only characters where some difference can be observed with a well-trained eye are the relatively shorter protibial and protarsi of the female versus those of the male counterpart, as well as the slight concavity of the abdominal sternites in the male.

#### Distribution.

**Figure 7.**
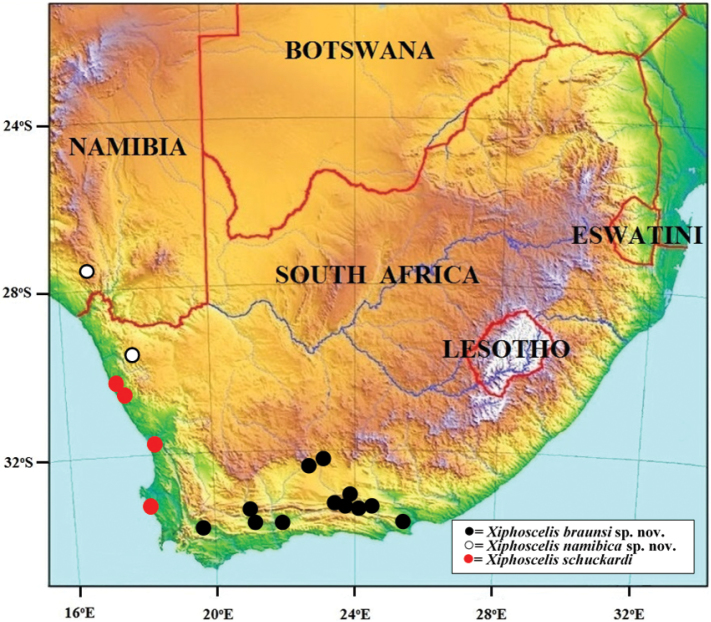
Known distribution range of *Xiphoscelis braunsi* sp. nov., *X. namibica* sp. nov. and *X. schuckardi* Burmeister, 1842 within southern Africa (Adapted from Mapsland: Copyright© 2019 Mapsland).

So far, the few specimens known for this species have been collected mostly in south-western Namibia, near the town of Rosh Pinah, in the Namuskluft area at about 1200 m asl (Fig. 7). One female specimen is also known from O’Kiep, in the South African Northern Cape. Thus, the species appears to be an arid mountain specialist, possibly occurring throughout the Ai-Ais Huns Mountains, the Richtersveld range and nearby areas above the Great Escarpment.

#### Biology.

The holotype and paratype series collected by Holm & Gebhardt in southern Namibia were all retrieved dead from middens of the southern harvester termite *Microhodothermes viator*. Given the very limited number of observations available for this species, it is not possible to establish whether or not this is a case of obligatory association, or again a rather opportunistic one.

#### Type material.

**Holotype** (♂): Namibia, Namuskluft, 1200 m, 27°45'S, 16°53'E, 2–6 Apr 2002, in *Microhodothermes viator* middens, E. Holm & H. Gebhardt (ISAM). **Paratypes**: 3 ♂, 2 ♀, same data as holotype (BMPC); 1♀, South Africa, Northern Cape, O’Kiep 29°35S, 17°52E, 1885-11-10, L. Péringuey leg. (SANC–COLS–12181).

### Updated identification key to the species of *Xiphoscelis* Burmeister, 1842

**Table:** 

1	Dorsal habitus completely black and shiny; elytral costae markedly elevated and visible; general dorsal sculpture dense and deep; body length 14–22 mm, larger than all other species; distribution: west coast lowlands of Western and Northern Cape	***X. shuckardi* Burmeister, 1842** (Fig. 3)
–	Dorsal habitus black or brown and matte or velutinous; elytral costae weakly elevated and dorsal sculpture scattered and shallow; body length 11–16 mm	**2**
2	Dorsal habitus exhibiting cretaceous markings	**3**
–	Body without cretaceous markings	**4**
3	Cretaceous ornamentation extensive on elytral surface, pronotal margins and protruding areas of abdominal sternites; body covered in long, scattered black setae; distribution: high mountains of Eastern Cape Karoo, above Great Escarpment	***X. sneeubergensis* Perissinotto, Villet & Stobbia, 2003** (Fig. 10)
–	Cretaceous markings moderately developed on pronotal margins and occasionally present also on elytra, but very faintly; body velutinous and covered in dense, medium to long golden-brown or orange setae; distribution: eastern areas of Eastern Cape Karoo	***X. lenxuba* Perissinotto, Villet & Stobbia, 2003** (Fig. 11)
4	Dorsal habitus completely black and matte; metatibial internal apical spine as long as spurs in both sexes; anterior clypeal margin weakly sinuate to straight; distribution: south-western Namibia and adjacent areas of Northern Cape	***X. namibica* Perissinotto, sp. nov.** (Fig. 2)
–	Dorsal habitus black or brown to reddish-brown; metatibial spurs and particularly inner apical spine hypertrophic in male; anterior clypeal margin strongly sinuate; distribution: Cape Fold Belt of the Western and Eastern Cape provinces	***X. braunsi* Perissinotto & Šípek, sp. nov.** (Figs 1, 8, 9)

**Figure 8.**
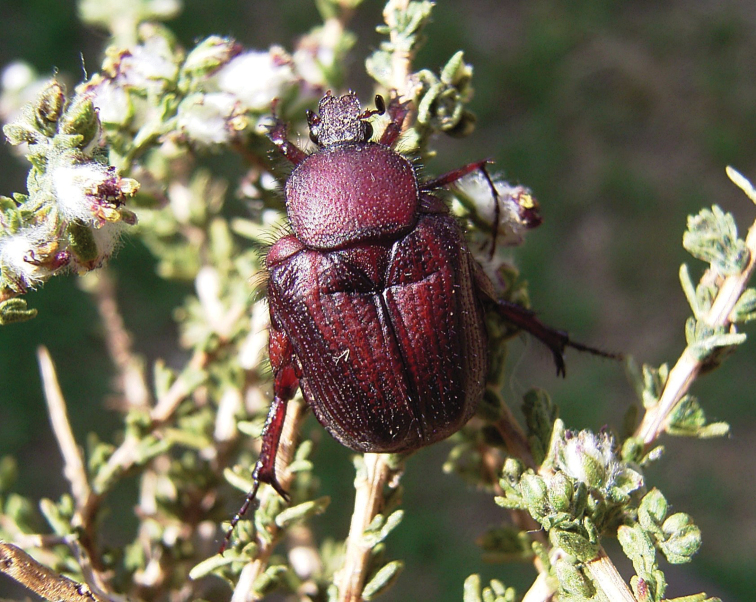
Reddish-brown female specimen of *Xiphoscelis braunsi* sp. nov. in its natural habitat at Willowmore, Dec 2005. Photograph by Lynette Clennell.

**Figure 9.**
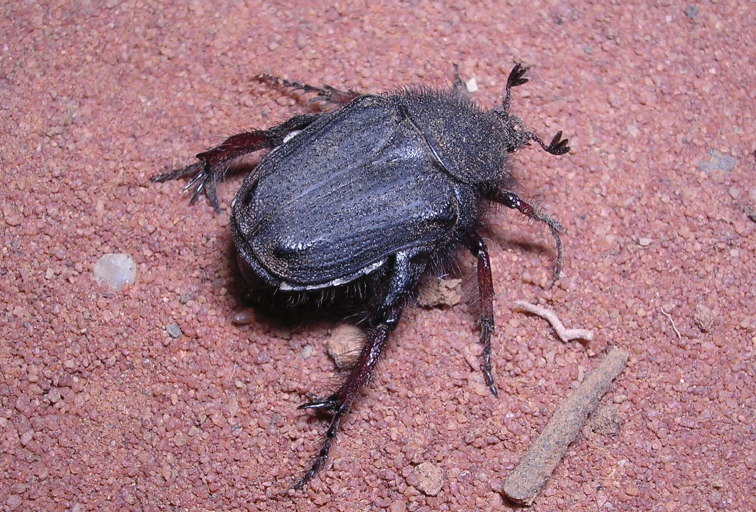
Black male specimen of *Xiphoscelis braunsi* sp. nov. in its natural habitat at Worcester, Sep 2017. Photograph by Mike Picker.

**Figure 10.**
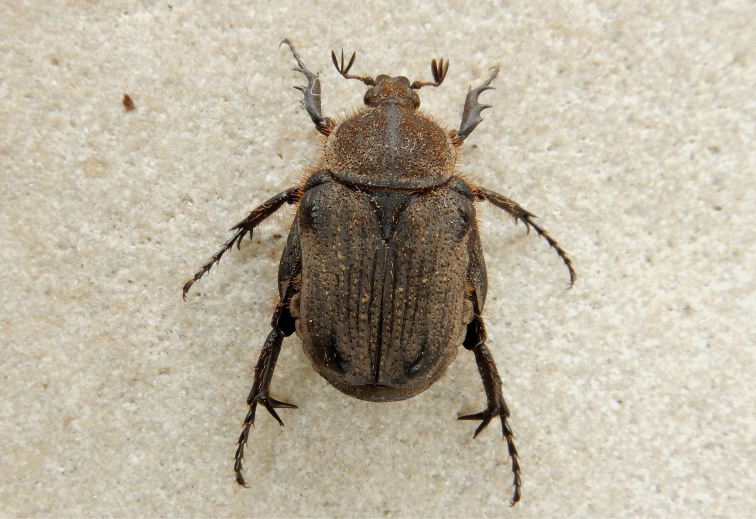
Male specimen of *Xiphoscelis lenxuba* Perissinotto, Villet & Stobbia, 2003 in its natural habitat in the Winterberg, Dec 2016. Photograph by Lynette Clennell.

**Figure 11.**
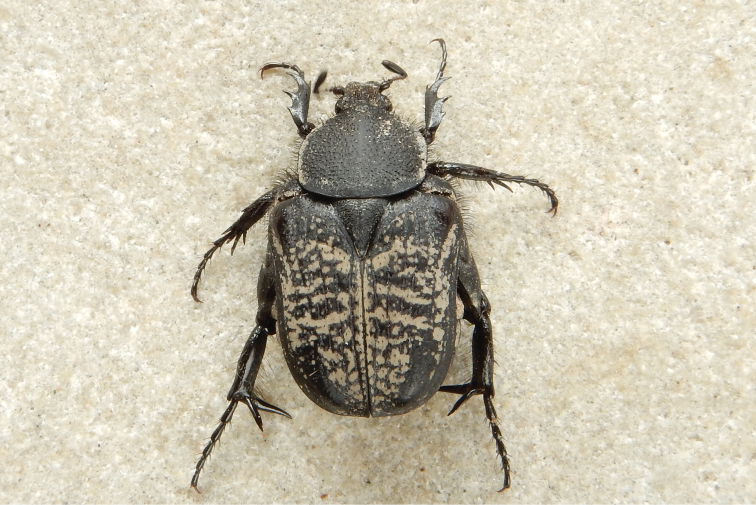
Male specimen of *Xiphoscelis sneeubergensis* Perissinotto, Villet & Stobbia, 2003 in its natural habitat in the Compassberg, Jan 2017. Photograph by Lynette Clennell.

### Larval morphology

#### Description of third instar larva of *X. braunsi* sp. nov.

Figs 12–14

**Differential diagnosis.** The larvae of *Xiphoscelis braunsi* sp. nov. are characterised by the following characters: long and prolific chaetotaxy on cranium; frons with 2–3 posterior frontal setae, anterior and lateral frontal setae long; epipharynx with sensorial cone (left nesium) desclerotised, low, obtuse, reduced and plate-shaped; mandibles with an external tooth on lateral margin; lacinial unci unequally fused at their base, smaller uncus about three times shorter than the longer one; hypopharyngeal scleroma without truncate process; pretarsi cylindrical with 11–12 setae, raster composed of 2 slightly subparallel rows of 14–20 pali, septula open posteriorly, narrow subtriangular to elliptical.

Larvae of *X. braunsi* sp. nov. differ from all known larvae of Cetoniinae*sensu stricto* by the absence of the hypopharyngeal truncate process. From those of *Meridioclita capensis* and *Heteroclita haworth* larvae of *X. braunsi* sp. nov. differ by the absence of the minute tip on the cylindrical pretarsi. Also, they can be separated from those of *Ichnestoma rostrata* by their only slightly emarginate frontal suture, by the trapezoidal clypeus and by the number of posterior frontal setae.

**Material studied.** Eight last instar larvae: EC, Sarah Baartman District (Dr Beyers Naudé Municipality), 15 km NW of Willowmore, 870 m, 5.I.2017. Fifteen larvae were collected in soil with organic debris under a shrub of *Psilocaulon* sp. (Mesembryanthemaceae), where also adult speciemens were found together with larvae. No termites or ants were observed in the vicinity of the shrub. The identity of the larvae has been confirmed by rearing the remaining specimens to adulthood and by molecular match (COI) with adult specimens.

**Figure 12.**
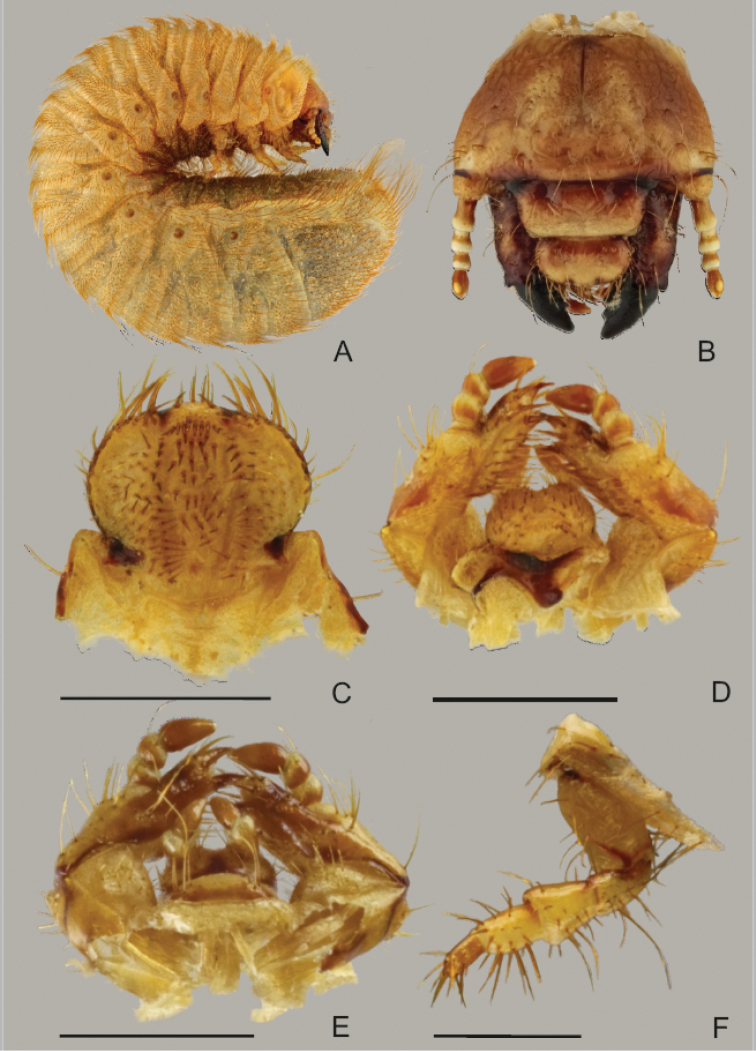
Last instar larva of *Xiphoscelis braunsi* sp. nov.: habitus (**A**), head capsule (**B**), epipharynx (**C**), maxillo-labial complex, dorsal view (**D**); maxillo-labial complex, ventral view (**E**), right metathoracic leg, anterior view (**F**). Scale bars: 1 mm.

***General body.*** Scarabaeiform (Fig. 12A); maximum length 30–35 mm; cranium yellowish-brown to brown, glabrous; body whitish with creamy shades; abdominal segments IX and X fused dorsally, ventrally separated by incomplete groove.

***Head capsule*** (Fig. 12B). Maximum width 2.7–3.1 mm; surface of cranium shiny and glabrous, exhibiting sparse microsculpture with few irregular crooked lines; cranium yellowish-brown to brown; antennifer, postclypeus, posterior half of labrum and frontoclypeal border dark brown; mandibles brown with black apices and central pale-brown area; cranial chaetotaxy as summarized in Table 1; setae generally abundant, with groups of posterior, lateral and dorsoepicranial setae almost fused; lateral epicranial setation with numerous medium and short setae and one prominent anterior long seta; frontal sutures lyriform or slightly bisinuate; epicranial insertions of antennal muscles almost indistinct; anterior and lateral frontal setae long; clypeus trapezoidal to subrectangular, anteclypeal area narrow (less than ¼ of entire clypeal area); postclypeus with one anterior and one pair of long lateral clypeal setae; stemmata present.

**Table 1.** Cranial chaetotaxy of the larva of *Xiphoscelis braunsi***sp. nov**. Abbreviations: AAS = setae of anterior frontal angle; ACS = anterior clypeal setae; AES = anterior epicranial setae; AFS = anterior frontal setae; DES = dorsoepicranial setae; LCS = lateral clypeal setae; LES = lateral epicranial setae; LFS = lateral frontal setae; PES = posterior epicranial setae; PFS = posterior frontal setae.

Group of setae	Epicranium				Frons				Clypeus	
	DES	PES	AES	LES	PFS	LFS	AFS	AAS	ACS	LCS
Long/medium setae	5–9	1–5	1	9–15	2–30	1	1	1	1	2
Minute/short setae	13–23	3	–	3–6	–	–	–	–	–	–

***Antennae*** (Fig. 12B). Tetramerous (an I–IV), relative length of antennomeres: an I > an IV > an II > an III; first antennomere (an I) about length of an II and an III combined; antennomere III with ventral, apical projection exhibiting single sensory spot; last antennomere (an IV) with one dorsal, three ventral sensory spots and one single round, apical sensory field.

***Labrum*** (Fig. 12B). Symmetrical, anterior margin trilobed with numerous setae; clithra present; dorsal surface of central part with two prominent setae on each side (one near labrum centre and one on lateral margin); posterior part of labrum with 1–3 medium sized setae on each side.

**Figure 13.**
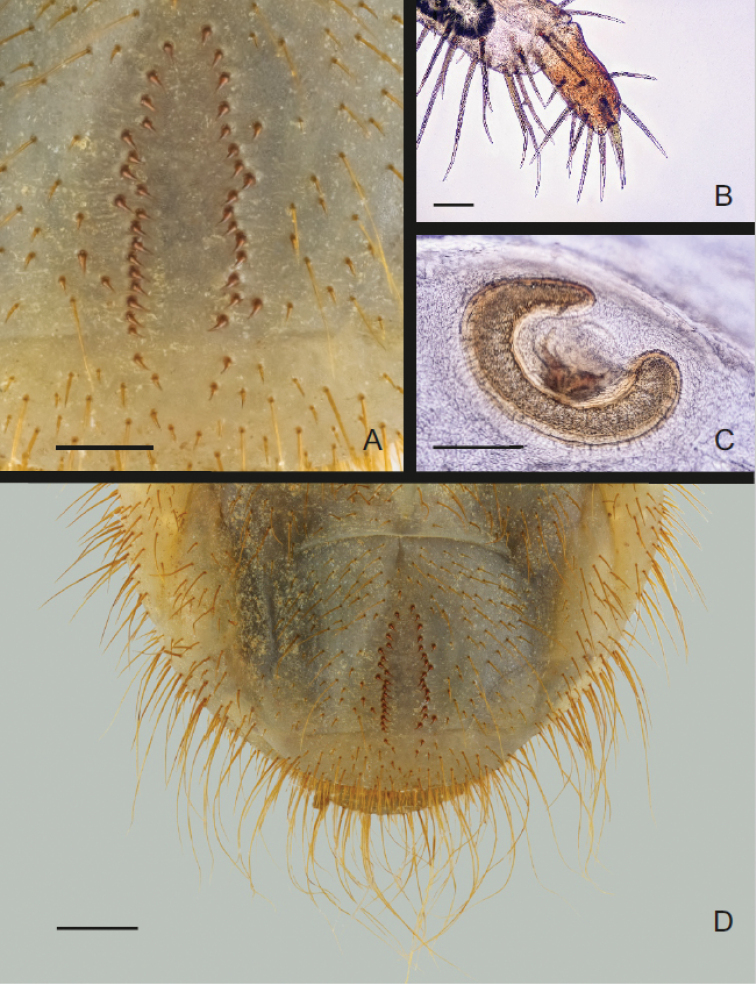
Last instar larva of *Xiphoscelis braunsi* sp. nov.: details of raster (**A**), right metapretarsus (**B**), thoracic spiracle (**C**) and general view of raster (**D**). Scale bars: 0.5 mm (**A**); 0.1 mm (**B, C**); 1 mm (**D**).

***Epipharynx*** (Figs 12C, 13A). Haptomerum exhibiting convex zygum with transverse, broadly arcuate row of approximately 8–9 stout setae and another 1–2 rows of 2–4 stout and prominent setae posteriorly to main row; anterior portion of zygum with aproximately 5–6 campaniform sensilla; haptomeral process and proplegmata absent; acroparia with medial labral lobe about half the size of lateral lobes; margin of medial labral lobe with 8 setae; lateral labral lobes with 8–10 setae; acanthoparia with 6–8 spine-like to hair-like setae, increasing in size from basal to apical part of acanthoparia; plegmata absent; chaetoparia asymmetric, right chaetoparia with 50–57 setae in total, left with 41–47 setae; chaetoparia with prominent row of 14–15 long and stout setae on each side; several more or less irregular rows of slender hair-like setae towards exterior margin of epipharynx; pedium covered with numerous irregular short but stout setae; gymnoparia absent; dexiotorma subtriangular, short; right pternotorma absent or heavily reduced; laeotorma short and narrow; left pternotorma triangular and large; haptolachus with sensorial cone (left nesium) desclerotised, low and obtuse with four pores, but lacking sclerotized plate (right nesium); plate-shaped sclerite, phoba and crepis absent.

***Mandibles*** (Fig. 13B–F). Asymmetrical, elongate and narrow, scissorial area about same length as molar area; scrobis with 9–13 setae, longitudinal furrow absent; external mandibular margin with prominent tooth-like projection in apical half; antero-lateral portion of dorsal mandibular surface with two prominent setae and 1–4 dorsomolar setae concealed in single rim on both mandibles; ventral surface with 4–5 ventromolar setae; stridulatory area with 27–32 transversal, uneven ridges; distance between distal ridges about twice as large as between proximal ones; left mandible with four scissorial teeth; second, third and fourth teeth of left mandible obtuse; right mandible with three well-developed scissorial teeth; distal molar lobe of left mandible oblique, dorsal end pointing strongly towards base of mandible; posterior margin of right madibular calyx bilobed in medial view, both lobes subtriangular; dorsal lobe about twice as large as ventral; calyx of left mandible flattened medially, lateral borders emarginate, posterior margin convex; brustia with 3–4 setae on both mandibles.

***Maxilla*** (Figs 12D–E, 15C–D). Dorsal surface of cardo with 9–12 setae, labacoparia with 16–24 setae; dorsomedial surface of stipes with 18–25 more or less slender hair-like setae, oblique row of 3–6 spine-like stridulatory teeth and anterior truncate process (blunt tubercle); postero-lateral part of stipes with 7–8 prominent long setae; ventral surface of stipes and mala brownish, setae prominent and mostly in desclerotised field; approximately 20 setae present on ventral surface of labacoparia; galea and lacinia entirely fused forming mala, galeo-lacinial suture indistinct, entirely absent on ventral surface; galear portion of mala with single falcate uncus and 5 long and stout hair-like setae in longitudinal rows, and 4 slender and shorter setae; lacinia with 15–20 long hair-like setae and two unequal unci fused at their base, smaller uncus about ¼ the length of the larger one; maxillary palps tetramerous, penultimate palpomere bearing two setae.

***Hypopharyngeal sclerome*** (Figs 12C, 15A). Asymmetrical, narrow; truncate process rudimental or absent; left lateral lobe with irregular arcuate row of 5–8 prominent setae, also two large setae proximal to right part of mesolateral margin of hypopharyngeal sclerome (remark: this structure is usually referred to as “tufts of tegumentary expansions” or “phoba”; however the SEM image shows a clear rim around each seta, Fig. 15A); both lateral lobes only feebly sclerotized with approximately 5–12 setae.

**Figure 14.**
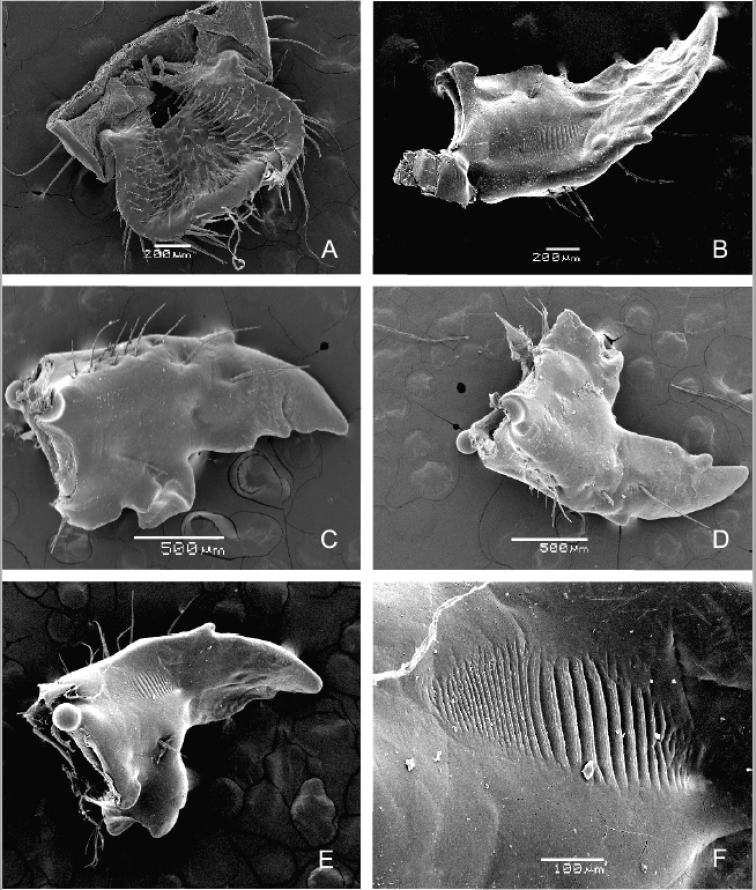
Last instar larva of *Xiphoscelis braunsi* sp. nov., SEM images: epipharynx (**A**); left mandible, ventral view (**B**); left mandible, dorsal view (**C**); right mandible, dorsal view (**D**); right mandible, ventral view (**E**); right mandible, ventral view - detail of stridulatory area (**F**).

**Figure 15.**
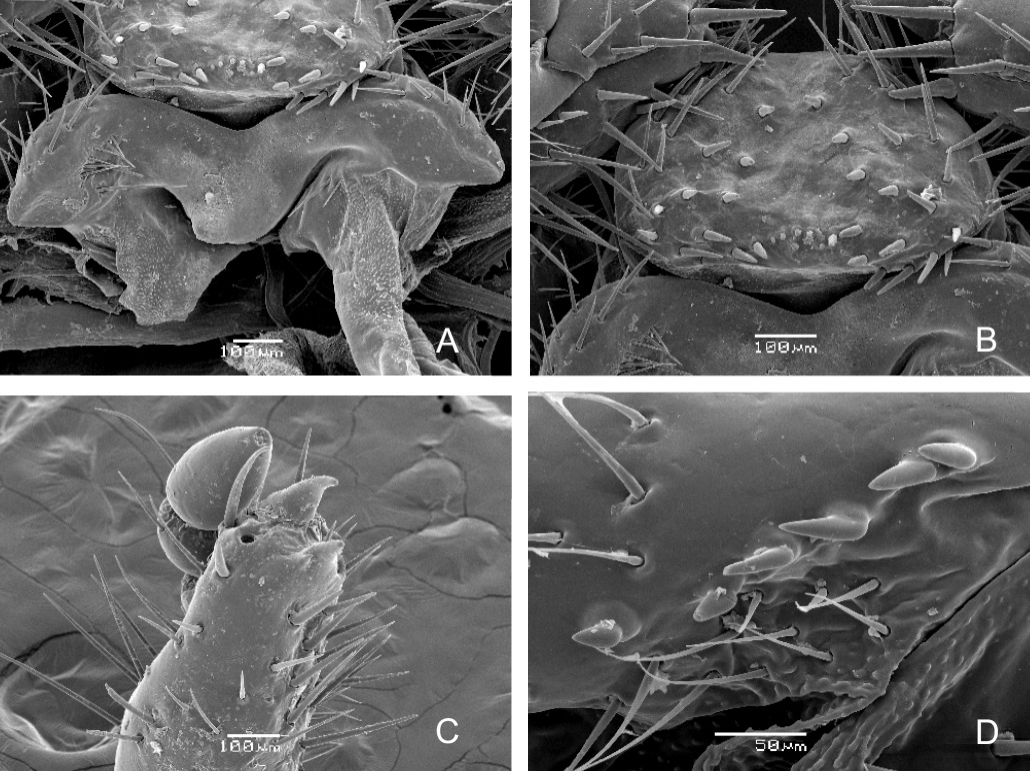
Last instar larva of *Xiphoscelis braunsi* sp. nov., SEM images: hypopharynx (**A**); ligula (**B**); mala with galear and lacinial unci (**C**); maxillar stridulatory teeth (**D**).

***Ligula*** (Figs 12C, 15B). Dorsal surface with approximately 40 prominent setae, medial and proximal setae stout, almost conical, but setae on distal part and on lateral margin long and hair-like; base of ligula with proximal arcuate or transverse row of approximately 8–10 campaniform setae; labial palpi bimerous; proximal sclerite of prementum with 4 long, prominent setae on each side.

***Thorax*** (Fig. 12A). Prothorax with single dorsal lobe, meso- and metathorax with three well-developed lobes; dorsi of each thoracic sublobe almost entirely covered with dense yellowish-brown setae, organized in approximately 3–6 rows; anterior rows with short setae, posterior row(s) with setae 3–5× longer; prothoracic sclerite well sclerotized, covering almost completely lateral portion of prothorax, bordered by dense line of setae; thoracic spiracle (Fig. 13C) with C-shaped respiratory plate; lobes of respiratory plate well separated with distance between lobes almost twice as large as maximum diameter of respiratory plate; respiratory plate with approximately 10–15 holes across diameter; bula with obtuse tubercle; all pairs of legs (Figs 12F) subequal in shape and size; pretarsi (Fig. 13B) cylindrical with 11–12 setae, claw absent.

***Abdomen*** (Figs 12A, 13A, D). Nine-segmented; dorsum of abdominal segments I–VI with 3 sublobes, segments VII and VIII with only 2; each sublobe bearing 3–4 rows of setae; setation of abdominal segments I–VIII similar to that of thorax; abdominal spiracles similar to mesothoracic spiracle, all spiracles subequal in size, bula with obtuse tubercle; dorsum of last abdominal segment (segments IX and X fused) evenly covered by dense medium and long setae; anal slit transverse.

***Raster*** (Fig. 13A, D). Palidium monostichous, with few irregular pali occasionally scattered around main row; raster composed of 2 slightly subparallel rows of 14–20 pali; pali with distal end dorsoventrally flattened distally, with pointed apices; septula open posteriorly, narrow subtriangular to elliptical, about 3 times longer than wide; tegilla sparsely setose but covering almost completely ventral surface of last abdominal segment; setae of tegilla and medium/long slender hair-like setae interspersed with few hamate setae and several long setae.

## Discussion

Apart from a number of characters that have led to believe that the genus *Xiphoscelis* may occupy a very primitive position in the phylogenesis of the Cetoniinae, species of the genus also exhibit a most unusual feature, represented by the extreme hypertrophy of structures in their metalegs. This is very prominent in males but less so in females and includes the femora, spurs and especially the inner spines, which are truly extraordinary in their thickness, length and sharpness in virtually all the species of the genus, possibly with the exception of *X. namibica* sp. nov. (Fig. 2 A–C).

Males of the largest species, *X. schuckardi* (Fig. 3 A–C), can inflict painful although superficial punctures on the skin of collectors when handled, by pushing through their metatibial inner spines with remarkable strength. This action generally escalates when gripping pressure is applied on their body (RP pers. obs, JB Ball & AP Marais pers. comm.). Such behaviour suggests a possible defence function for this apparatus, which may be used quite effectively against the various ground predators that occur in their natural habitat, such as lizards, agamas, geckos, frogs and toads as well as birds. Apart from this defence function, both inner spines and spurs are also involved in reproduction, as males have been observed exerting enhanced grip on females during mating using this apparatus (RP pers. obs.).

As reported earlier, all species of the genus are restricted to the south-western arid and semi-arid regions of southern Africa (Holm and Marais 1992, Perissinotto et al. 2003). The two species described in a previous revision by Perissinotto et al. (2003), *X. lenxuba* (Fig. 10) and *X. sneeubergensis* (Fig. 11), seem to inhabit exclusively the mountainous region of the eastern Karoo, with the first occurring from the upper reaches of the Great Fish River Valley to the interior of the Winterberg range at altitudes < 1000 m, in the Albany Thicket Biome. On the other hand, *X. sneeubergensis* is found at altitudes of 1500–2000 m asl in the Sneeuberg and Bamboesberg ranges (Perissinotto et al. 2003, RP pers. obs.), in the upper bioregion of the Nama-Karoo Biome (Mucina and Rutherford 2006) .

Following the description of the two new species here, it is evident that *X. schuckardi* is restricted to the lowlands of the South African west coast, from just north of Cape Town to the Namaqualand area of the Northern Cape (Fig. 7). The typical vegetation biome here can be classified as ranging from Fynbos of the Western Strandveld type to Succulent Karoo of the Namaqualand Sandveld type (Mucina and Rutherford 2006). *Xiphoscelis braunsi* sp. nov. appears to be distributed across the entire belt of the Cape Fold Mountains, but at altitudes generally not exceeding 1000 m asl, in a wide variety of bioregions within the Succulent Karoo or Fynbos biomes. *Xiphoscelis namibica* sp. nov. inhabits the arid region of the Huns Mountains in Namibia and nearby ranges in the Northern Cape Province of South Africa, possibly including the Richtersveld (Fig. 7), in the Desert to Succulent Karoo biomes (Mucina and Rutherford 2006).

The frequently reported association with the termite *Microhodothermes viator* (Péringuey 1907, Holm and Marais 1992, Holm and Stobbia 1995), appears to apply only to the three species previously grouped under *X. schuckardi* (Perissinotto et al. 2003). However, at least for *X. braunsi* sp. nov. (Figs 1, 8, 9) this is not an obligatory association, as on several occasions both larvae and adults were found in detrital accumulations of non-termite origin, including leaf litter and aardvark dung (RP pers. obs.). Thus, larvae of this species (and possibly others in the genus) may actually exploit a suite of food sources as part of an opportunistic strategy aimed at utilising any accumulation of decomposing matter in their dry and resource-scarce habitats.

The Xiphoscelidini, or more accurately Xiphoscelidina (refer to Šípek and Král 2012 for further details), have been traditionally regarded as one of the most “primitive” or “basal” group of the Cetoniinae. Holm and Marais (1992) considered the genus *Xiphoscelis* as the “*Archaeopteryx*” among the fruit chafers. However, as pointed out previously, evidence in support of the Xiphoscelidina as an early clade in the cetoniine phylogeny has not been provided yet through any unambiguous morphological character. The latest molecular phylogenetic analysis of the Cetoniinae (Šípek et al. 2016) has questioned the classical interpretation of cetoniine phylogeny, suggesting a para- or polyphyletic structure for most of the tribes. At the same time, it has brought more insight into the intricate relationships within this group. In this analysis, the representatives of the tribes Osmodermatini, Taenioderini and Schizorhinini were identified as basal Cetoniinae “*sensu stricto*”. In other words, these groups have been identified as sister clades to the remaining clades, with the exception of the Trichiini and Valgini. Unfortunately, none of the putative representatives of Xiphoscelidina was present in the dataset.

**Figure 16.**
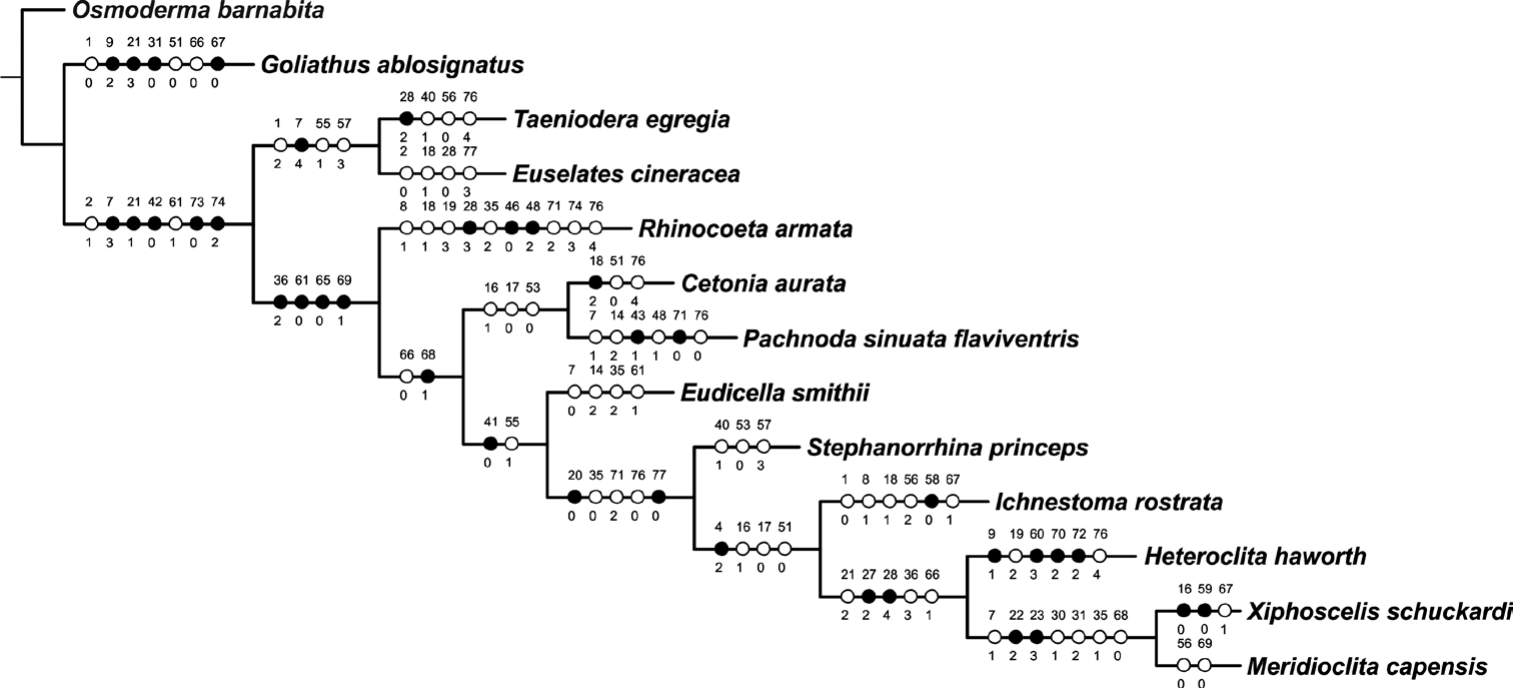
Strict consensus topology of 13 representatives of the Cetoniinae, based on a larval morphology dataset. Character states are marked on clades, with character number above each circle and state number below it; black circles indicate unique evolutionary events, while white circles denote reversals or parallelisms. Partitioned Bremer Support (PBS) values above 50% are indicated. The analysis was performed in order to highlight morphological similarities among representatives of distinct cetoniine clades.

Based on the description of the larval morphology of *X. braunsi*, we can conclude that the larvae of the genus *Xiphoscelis* are characterised by a remarkable subset of morphological characters: i.e., long and dense chaetotaxy of cranium; reduction of sense cone and plate-shaped sclerite of epipharynx; presence of an external tooth on lateral mandibular margin; shape and size of lacinial unci; shape of hypopharyngeal scleroma (especially the reduction of the truncate process); and shape of pretarsi. To allow an easy comparison of the morphological similarities of *Xiphoscelis* with other Cetoniinae larvae, a parsimony analysis of the larval morphology of 13 cetoniine genera, including *Xiphoscelis*, was performed (Fig. 16). *Osmoderma barnabita* Motschulsky, 1845 was used as an outgroup to root the tree and representatives of the tribes Cetoniini, Goliathini, Taenioderini were included in the analysis along with representatives of other “enigmatic”genera from the southern part of Africa, such as *Rhinocoeta cornuta* (Fabricius, 1781), *Meridioclita capensis* Krikken, 1982 and *Heteroclita haworth* (Gory & Percheron, 1833).

The outcome shows that the larva of *X. braunsi* sp. nov. falls within a clade shared with *M. capensis* and *H. haworth* (Fig. 16). This clade is supported by the following key synapomorphies: 1) presence of long posterior frontal setae; and 2) absence of plate-shaped sclerite. *Meridioclita capensis* is identified as the sister species of *X. braunsi*, based on the prolific cranial chaetotaxy and the absence or wide reduction of right pretnotorma and laeotorma. *Ichnestoma rostrata* Janson, 1878 is identified as a sister species to the clade comprised of all the above-mentioned species based on cranial chaetotaxy, number of dorsal and ventral sensory spots on antenna and presence of a conspicuous outer tooth on lateral mandibular margin. These results question the hypothetical “basal” placement of *Xiphoscelis* among the Cetoniinae (e.g., Holm and Marais 1992, Perissinotto et al. 2003).

In the early taxonomic classifications of the Cetoniinae, the genera *Xiphoscelis*, *Ichnestoma*, *Heteroclita* and *Meridioclita* were all classified under the Xiphoscelidini (e.g., Burmeister 1842, Schenkling 1921). However, at least some of the alleged larval apomorphies may indeed represent the result of convergent evolution forced by the arid and seasonal conditions of their habitat as well as the soil-dwelling habits of their larvae. Unfortunately, the recent attempt by Kouklík (2017) to resolve the phylogenetic placement of *Xiphoscelis* and other Xiphoscelidini genera on the basis of molecular DNA analysis has generated contradictory results. More efforts are, therefore, needed in order to unravel the true phylogenetic relationships within this clade.

## Supplementary Material

XML Treatment for Xiphoscelis braunsi

XML Treatment for Xiphoscelis namibica

## Appendix 1

Character states of the 77 morphological characters used in the analysis of larval similarity of 13 Cetoniinae genera (refer also to Fig. 16).

**1. Color of cranium**: 0 – black to dark brown, 2 – brown, 3 – light brown to yellowish

**2. Surface of cranium**: 0 – smooth or with very fine microsculpture, 1 – light to medium microsculpture, 2 – with coarse microsculpture

**3. Texture of cranial microsculpture**: 0 – more or less pitted, 1 – more or less linear

**4. Chaetotaxy of cranium**: 0 – light (short and/or very sparse setae), 1 – “standard Cetoniinae” type, 2 – dense (setae numerous and/or extraordinary long)

**5. Anterior end of epicranial suture**: 0 – originating at posterior end of frontal sutures, 1 – extending into frons, 2 – irregular, feebly visible

**6. Epicranial suture**: 0 – distinct, 1– indistinct

**7. Frontal suture**: 0 – straight, 1 – straight, slightly emarginate, 2 – convex, 3 – concave, 4 – bisinuate

**8. Frontal suture**: 0 – distinct, 1 – indistinct

**9. Posterior angle of frontal sutures**: 0 – acute, 1 – right, 2 – obtuse

**10. Widest part of cranium**: 0 – at base of antennae, 1 – at approx. one-third of cranium, 2 – at middle of cranium

**11. Number of antennomeres**: 0 – 3, 1 – 4, 2 – 5

**12. Sensory spot on third antennomere**: 0 – present, 1 – absent

**13. Third antennomere**: 0 – with well-developed ventral and apical projection, 1 – ventroapical projection only feebly developed, 2 – ventroapical projection absent

**14. Stemmata**: 0 – present and pigmented, 1 – present without pigment, 2 – reduced, 3 – absent

**15. Setae on antennomeres**: 0 – present (at least on a single joint), 1 – absent

**16. Number of sensory spots on dorsal side of antenna**: 0 – 1, 1 – 2 or 3, 2 – 4 or more, 3 – 1 or 2 (if both character states 1 and 2 are present)

**17. Number of sensory spots on ventral side of antenna**: 0 – 0 to 3, 1 – 4 and more, 2 – 3 to 5 (if both character states 1 and 2 are present)

**18. Shape of clyepus**: 0 – trapezoidal, 1 – rectangular

**19. Preclypeus**: 0 – about ½ of area of entire clypeus, 1 – about 1/3 of area of entire clypeus, 2 – about ¼ of area of entire clypeus, 3 – less than ¼ of area of entire clypeus

**20. Anterior and lateral frontal setae**: 0 – medium long to long, 1 – minute, 2 – absent

**21. Posterior frontal setae**: 0 – absent or minute, 1 – one medium long to long seta, 2 – 2 or more medium to long setae, 3 – both long and short setae

**22. Number of long lateral epicranial setae**: 0 – absent, 1 – 1 or 2, 2 – 3 or more

**23. Dorso-epicranial setae**: 0 – 1 to 3 medium to long setae together with some minute setae, 1 – only minute setae, 2 – only medium to long setae, 3 – both long and short setae in abundance

**24. Shape of anterior labral margin**: 0 – asymmetric, 1 – slightly convex, 2 – strongly convex, 3 – slightly concave, 4 – trilobed

**25. Shape of sensory cone (second nesium)**: 0 – ovoid, 1 – conical, 2 – not applicable

**26. Size of sensory cone (second nesium)**: 0 – well developed, 1 – more or less reduced, 2 – absent, indistinct

**27. Size of third nesium (plate-shaped sclerome)**: 0 – largely reduced, 1 – feebly sclerotized, reduced, 2 – absent

**28. Shape of third nesium (plate-shaped sclerome)**: 0 – transverse, 1 – present on right and in front of sensory cone (shape of upturned and reversed capital letter “L”), 2 – present on left side of sensory cone, 3 – present in front and on both sides of the sensory cone (shape of reversed capital letter “U”), 4 – not applicable

**29. Dexiotorma**: 0 – straight and wide, 1 – bent at inner end, 2 – straight, narrow, 3 – reduced

**30. Right pternotorma**: 0 – present, 1 – absent

**31. Laeotorma**: 0 – present, 1 – reduced, 2 – only feebly developed

**32. Left pternotorma**: 0 – present, 1 – absent

**33. Number of sensilla on haptolachus**: 0 – four, 1 – five, 2 – six or more

**34. Clithra**: 0 – absent, 1 – only single clithrum present, 2 – two clithra present

**35. Setae of acanthoparia**: 0 – directly on lateral margin, with swollen base, 1– directly on lateral margin, without distinctly swollen base, 2 – away from margin and slightly towards center of epipharynx

**36. Pedium**: 0 – absent, 1 – reaching < 25% of central part of epipharynx, 2 – reaching 25–50% of central part of epipharynx, 3 – reaching 51–75% of central part of epipharynx, 4 – reaching > 75% of central part of epipharynx

**37. Chaetoparia of epipharynx**: 0 – reaching only to level of laeo- and pternotorma, 1 – extending posteriorly behind level of laeo- and pternotorma

**38. Crepis**: 0 – well developed, 1 – reduced, 2 – absent

**39. Zygum**: 0 – flat or transverse, 1 – high and conical, 2 – extending into tylus, 3 – absent

**40. Sensilla of zygum**: 0 – not grouped on projection, 1 – grouped on distinct projection, 2 – intermediate

**41. Number of setae on zygum**: 0 – six, 1 – seven, 2 – eight and more, 3 – intermediate

**42. Transverse row of prominent setae on zygum**: 0 – arcuate, 1 – right angled, 2 – angulate, 3 – not applicable

**43. Setae at inner margin of zygum**: 0 – approximately of same size and shape as setae in prominent row, 1 – different from setae in transverse row, 2 – not applicable, 3 – absent

**44. Setae of chaetoparia**: 0 – all setae of approximately same size and shape, 1 – chaetoparia with several rows of longer and stouter setae

**45. Number of teeth on scissorial area of left mandible**: 0 – two, 1 – three, 2 – four

**46. Last two teeth of left mandible**: 0 – fused, 1 – separated

**47. Number of teeth on scissorial area of right mandible**: 0 – two, 1 – thee, 2 – four

**48. Second and third teeth of left mandible (number from the apex)**: 0 – separated, 1 – fused, 2 – not applicable

**49. Mandibular teeth**: 0 – in single plain, 1 – second and third tooth oriented dorsoventrally

**50. Dorsomolar setae**: 0 – absent on both mandibles, 1 – absent on left mandible, 2 – present on both mandibles

**51. Tooth on external mandibular margin**: 0 – present, 1 – absent

**52. Ventromolar setae**: 0 – present on both mandibles, 1 – absent on both mandibles

**53. Mandibular shape**: 0 – falcate at least at apex, 1 – more or less straight

**54. Mandibular stridulatory area**: 0 – well developed with transverse ridges on entire area, 1 – developed, ridges only feebly developed, 2 – absent

**55. Shape of stridulatory area**: 0 – oval, 1 – oblong, 2 – intermediate

**56. Number of stridulatory ridges on mandible**: 0 – less than 10, 1 – 10 to 20, 2 – 20 or more, 3 – intermediate

**57. Extent of ventral asperities**: 0 – reaching towards ventral process of mandibles, 1 – not reaching towards ventral process of mandibles, 2 – reaching towards ventral process only on left mandible, 3 – reaching towards ventral process only on right mandible

**58. Shape of ventral asperities (obr. 17)**: 0 – small tubercle, 1 – tooth-like process

**59. Hypopharyngeal scleroma**: 0 – symmetrical, without processes, 1 – asymmetric, with large process on right side

**60. Number of maxillar stridulatory teeth**: 0 – 0, with indistinct transverse ridges, 1 – 4 to 9, reduced, 2 – 4 to 9, well developed, 3 – 10 to 20

**61. Shape of stridulatory teeth**: 0 – short, with swollen base, 1 – long and sharp, 2 – obtuse tubercles, 3 – more than one type, 4 – intermediate shape

**62. Lacinia and galea**: 0 – fused, forming mala, 1 – separated

**63. Number of lacinial unci**: 0 – three, 1 – two, 2 – one or none

**64. Shape of legs**: 0 – short and stout, 1 – slender and long

**65. Relative length of legs**: 0 – each pair increasing in size from protorax to metathorax, 1 – all pairs (sub)equal

**66. Legs: relative length of tibiotarsus and trochanter joints**: 0 – trochanter longer than pretarsus, 1 –- trochanter shorter than pretarsus

**67. Shape of pretarsus**: 0 – falcate, pointed with pair of setae, 1 – conical, setae in apical half, 2 – conical, with numerous setae and small claw on apex, 3 – narrow, pointed and slightly bent, with swollen base and a pair of basal setae, 4 – almost reduced with pair of setae

**68. Relative size of pretarsi**: 0 – all pairs equal in length, 1 – increasing in size from prothorax to metathorax, 2 – decreasing in size from prothorax to metathorax.

**69. Tibiotarsi**: 0 – all pairs equal in shape and size, 1 – first two pairs similar, last pair of different size, 2 – all three pairs same in size but of different shape

**70. Size of spiracles**: 0 – all spiracles (sub)equal, 1 – first or first and second abdominal spiracles distinctly smaller than thoracic spiracles, 2 – size decreasing posteriorly

**71. Shape of thoracic spiracles**: 0 – concealed, arms of respiratory plate almost closed, 1 – open but opening between arms narrow, 2 – open with arms of spiracular plate well separated

**72. 9^th^ and 10^th^ abdominal segments**: 0 – entirely fused, 1 – separated, 2 – partially fused

**73. Palidium**: 0 – present, 1 – absent

**74. Size of Palidium**: 0 – absent, 1 – short, 2 – of medium length, reaching middle of last abdominal segment, 3 – long, reaching almost end of last abdominal segment

**75. Hammate setae**: 0 – present, 1 – absent

**76. Setae of last abdominal segment (excluding raster)**: 0 – long, hair-like, 1 – short, 2 – long, dorsoventrally flattened at distal margin, 3 – absent, 4 – mixture of long hair-like and short stout setae

**77. Chaetotax (general appearance) of larva**: 0 – hairy, with numerous long setae. 1 – dense, covered in numerous short setae, 2 – standard “Cetoniinae” type, 3 – sparse
